# Redox Biology and Insulin-like Growth Factor-Binding Protein-6: A Potential Relationship

**DOI:** 10.3390/biology14070747

**Published:** 2025-06-23

**Authors:** Anna Rita Daniela Coda, Arcangelo Liso, Francesco Bellanti

**Affiliations:** 1 C.R.E.A.T. E—Center for Research and Innovation in Medicine, Department of Medical and Surgical Sciences, University of Foggia, 71122 Foggia, Italy; daniela.coda@unifg.it; 2 Department of Medicine and Surgery, University of Perugia, 06123 Perugia, Italy; arcangelo.liso@unipg.it

**Keywords:** IGFBP-6, redox homeostasis, immune regulation, fibrosis

## Abstract

Insulin-like growth factor-binding protein 6 (IGFBP-6) is primarily recognized for its inhibitory effects on insulin-like growth factor 2 (IGF-2), regulating processes such as cell proliferation, differentiation, and survival. However, recent studies have revealed that IGFBP-6 also participates in a range of IGF-independent activities, notably in redox biology, immune regulation, and fibrosis. These IGF-independent actions involve interactions with redox-sensitive signaling pathways that influence mitochondrial metabolism, neutrophil function, and fibroblast activity, all of which are central to redox-dependent processes in inflammation and fibrosis. Despite these insights, the precise mechanisms by which IGFBP-6 modulates redox signaling remain largely unexplored. This review examines the growing understanding of IGFBP-6 beyond its classical role as an IGF-binding protein, with a focus on its involvement in redox homeostasis. By exploring these emerging roles, we aim to elucidate how IGFBP-6 contributes to redox homeostasis and to assess its potential as a therapeutic target in oxidative stress-related diseases, including fibrosis, cancer, and immune dysfunction.

## Simple Summary

Insulin-like growth factor-binding protein 6 (IGFBP-6) is a protein that typically regulates insulin-like growth factor 2 (IGF-2), which controls important cellular processes like growth and survival. However, recent studies have shown that IGFBP-6 also has IGF-independent roles, particularly in regulating redox biology and influencing immune responses and tissue repair. Redox homeostasis, which involves balancing reactive species (RS) and antioxidants, is crucial for maintaining normal cell function. Disruptions in this balance can lead to various diseases, such as cancer, fibrosis, and neurodegeneration. IGFBP-6 has been shown to interact with redox-sensitive pathways, affecting oxidative stress responses and immune cell activation. Despite the growing evidence of its involvement in redox biology, the exact mechanisms through which IGFBP-6 influences redox homeostasis remain unclear. This review explores the emerging role of IGFBP-6 in redox regulation and its potential as a therapeutic target for diseases related to oxidative stress and fibrosis.

## 1. Introduction

The insulin-like growth factor (IGF) system is a well-established regulatory network that governs critical cellular functions, including growth, metabolism, and survival. It consists of IGF-1, IGF-2, their receptors, and a family of six insulin-like growth factor-binding proteins (IGFBPs) that modulate IGF activity [1]. Among these, IGFBP-6 stands out for its preferential binding to IGF-2, effectively inhibiting its biological actions [2]. However, growing evidence suggests that IGFBP-6 also exerts significant IGF-independent effects, indicating a broader role in cellular physiology [3].

One of the most intriguing developments in recent years is the proposed involvement of IGFBP-6 in redox biology. Redox homeostasis, the balance between reactive species (RS) and antioxidants, plays a central role in maintaining cellular function. Under normal physiological conditions, low-to-moderate levels of RS, such as reactive oxygen species (ROS) and reactive nitrogen species (RNS), serve as essential signaling molecules involved in immune responses, cell differentiation, and metabolism [4,5]. However, when this balance is disrupted, excess RS can lead to oxidative stress, a condition implicated in a wide range of diseases, including cancer, neurodegenerative disorders, cardiovascular diseases, and fibrosis [6]. A growing body of research highlights the role of redox signaling in various cellular processes, including inflammation, apoptosis, and tissue repair [7]. While the roles of classical redox regulators and signaling pathways are well-characterized, the contribution of non-canonical players like IGFBP-6 remains poorly understood.

Current evidence suggests that IGFBP-6 may influence redox-sensitive processes such as immune cell activation, oxidative stress response, and fibroblast proliferation. Notably, IGFBP-6 has been linked to mitochondrial metabolism and neutrophil oxidative burst, processes central to inflammation and tissue repair [8]. Yet, despite these associations, the underlying molecular mechanisms connecting IGFBP-6 to redox regulation are still largely unexplored.

Given the emerging relevance of IGFBP-6 in redox signaling and its implications in immune modulation and fibrosis, this review aims to synthesize current knowledge, identify gaps, and propose potential directions for future research. Understanding how IGFBP-6 interfaces with redox biology may open new avenues for therapeutic strategies targeting oxidative stress-related diseases.

## 2. Overview on Redox Biology and Signaling

### 2.1. Redox Homeostasis and Its Importance in Cellular Functions

Redox biology centers around the study of RS, particularly ROS and RNS, and their roles in cellular signaling and the maintenance of cellular homeostasis. Redox homeostasis refers to the balance between the production of these reactive species and the body’s ability to neutralize them with antioxidants [9]. This balance is critical for maintaining normal cellular functions such as metabolism, signaling, immune responses, and cellular growth [7]. Under physiological conditions, ROS are essential secondary messengers in signaling pathways that regulate important cellular processes like cell differentiation, proliferation, and apoptosis. However, when the production of RS overwhelms the antioxidant defense mechanisms, oxidative stress occurs, leading to cellular damage and contributing to a wide range of diseases, including cancer, cardiovascular diseases, neurodegenerative disorders, and fibrosis [10].

RS are produced through both endogenous and exogenous sources. Endogenously, the primary source of RS is mitochondrial oxidative phosphorylation, during which oxygen is reduced in the electron transport chain, resulting in the production of superoxide (O_2_^−^) [11]. Other sources of RS include NADPH oxidases (NOXs), xanthine oxidase, and lipoxygenases [12,13]. Exogenous factors, such as ultraviolet (UV) radiation, pollutants, and toxins, can also lead to an increased generation of RS [14]. While RS acts as crucial signaling molecules at low concentrations, excessive RS accumulation results in oxidative damage to proteins, lipids, and DNA, which can disrupt cellular components and lead to aging, genetic mutations, tumorigenesis, and other chronic diseases. This dual role of RS, acting both as signaling molecules and agents of cellular damage, emphasizes the delicate balance that must be maintained for cellular health [14,15,16].

### 2.2. Antioxidant Mechanisms and Redox Signaling

To counterbalance the harmful effects of RS, cells utilize various antioxidant defense systems. These include both enzymatic and non-enzymatic antioxidants. Enzymatic antioxidants such as superoxide dismutase (SOD), catalase, and glutathione peroxidase help neutralize RS by converting them into less reactive species [17]. For instance, SOD catalyzes the conversion of superoxide to hydrogen peroxide (H_2_O_2_), which is then reduced to water by catalase or glutathione peroxidase [18]. Non-enzymatic antioxidants, including glutathione, vitamin C, and vitamin E, also play vital roles in scavenging RS and preventing oxidative damage [19]. Additionally, cellular systems like the thioredoxin and glutaredoxin pathways are involved in regulating the oxidation states of proteins, which are critical for maintaining redox signaling and cellular function [20]. A disruption in these antioxidant systems can lead to a build-up of RS, contributing to diseases like neurodegeneration, cardiovascular disorders, cancer, and fibrosis [21,22]. Therefore, maintaining the delicate balance between RS production and antioxidant defense is vital for cellular health and overall well-being.

Redox signaling is integral to many physiological processes, including immune responses, cell cycle regulation, apoptosis, and autophagy. RS play a critical role in activating redox-sensitive transcription factors such as the nuclear factor-kappa B (NF-κB) and the activator protein-1 (AP-1), which regulate immune responses, inflammation, and cell survival [23]. In immune cells, RS are crucial for the oxidative burst that occurs during the immune response, helping neutrophils and macrophages to clear pathogens [24]. Additionally, RS activate signaling pathways like the Ras/mitogen-activated protein kinase (MAPK) pathway, which regulates cell growth, survival, and apoptosis [25]. In cancer, RS can drive tumorigenesis by promoting oncogenic signaling and genetic mutations, whereas excessive RS levels can also induce tumor suppressor pathways that trigger cell death [26]. This complex balance between pro-tumorigenic and tumor-suppressive effects is influenced by the cellular redox environment. Furthermore, RS signaling contributes to the development of fibrotic diseases by activating pathways like tumor growth factor-β (TGF-β), which promotes fibroblast activation and extracellular matrix (ECM) deposition, leading to tissue scarring [27]. Understanding how RS regulate these processes is crucial for developing targeted therapeutic strategies to address oxidative stress-related diseases.

### 2.3. Redox Dysregulation, Reductive Stress, and Disease Implications

**Figure 1:**
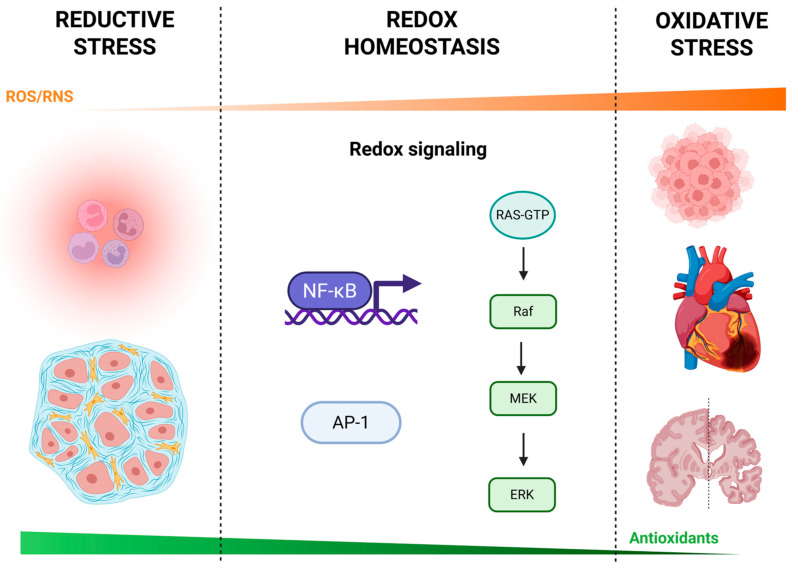
Continuum of reductive stress, redox homeostasis, and oxidative stress. A schematic overview of cellular redox biology illustrating how varying levels of reactive oxygen and nitrogen species (ROS/RNS) influence physiological and pathological outcomes. Reductive stress (**left**): low ROS/RNS levels can lead to immune suppression (e.g., impaired neutrophil function) and altered stromal cell activity, potentially contributing to tissue dysfunction. Redox homeostasis (**center**): balanced ROS/RNS engage redox-sensitive signaling pathways—including nuclear factor κB (NF-κB), activator protein-1 (AP-1), and the RAS–RAF–MEK–ERK (extracellular signal-regulated kinase) kinase cascade—that regulate gene expression, cell survival, and adaptive responses. Oxidative stress (**right**): excessive ROS/RNS overwhelm antioxidant defenses and drive inflammation, tumorigenesis, cardiovascular injury, and neurodegeneration. The orange gradient above indicates relative ROS/RNS levels, while the green gradient below reflects the capacity of endogenous antioxidants (e.g., superoxide dismutase, catalase, glutathione peroxidase, thioredoxin, glutaredoxin, glutathione, vitamins C and E). Created with BioRender.com.

Redox dysregulation, where there is an imbalance between the production of RS and the body’s antioxidant defenses, contributes to various pathologies (Figure 1).

While oxidative stress, due to excessive ROS, has been implicated in cancer, cardiovascular diseases, and neurodegenerative disorders, another critical form of dysregulation is reductive stress. Reductive stress arises when there is an excess of reducing equivalents such as NADPH and antioxidants, leading to an over-reduction of cellular components [28]. This imbalance impairs the oxidation of essential proteins, disrupting redox-dependent signaling and cellular functions like differentiation, apoptosis, and immune responses [29]. In cancer, reductive stress has been linked to enhanced tumorigenesis by preventing normal cell differentiation and promoting a more proliferative, undifferentiated phenotype [30]. In insulin resistance and diabetes, reductive stress interferes with glucose metabolism and disrupts cellular responses to insulin, contributing to metabolic dysfunction [31].

The interplay between oxidative and reductive stress plays a critical role in fibrosis, where both forms of redox imbalance contribute to the development and progression of tissue scarring. Oxidative stress triggers inflammation and tissue damage, which are key drivers of fibrosis, while reductive stress hinders normal inflammatory resolution and cellular repair mechanisms, potentially exacerbating fibrotic responses [32,33]. In liver fibrosis, for instance, a disrupted redox environment supports the activation of profibrotic signaling pathways like TGF-β, which drives excessive ECM production [34]. Understanding how oxidative and reductive stress both influence fibrotic progression highlights the need for therapeutic approaches that target redox homeostasis to mitigate chronic disease states associated with fibrosis.

## 3. Redox Modulation of Immunity, Tissue Repair, and Fibrosis

### 3.1. Redox Regulation in Innate and Adaptive Immunity

Scientific evidence highlights the critical role of oxidative and reductive stress in immune system modulation, influencing both innate and adaptive immunity. Autoimmune diseases, where the immune system attacks the body’s tissues, are often characterized by autoantibodies or autoreactive T cells that drive the loss of self-tolerance. Immune responses to viral or bacterial infections and tissue damage rely on the immune-mediated production of RS, with infections and environmental factors exacerbating disease onset [24]. Important immune pathways involved in RS production include tumor necrosis factor (TNF) and the NLR pyrin domain-containing 3 (NLRP3)/caspase-1 inflammasome, which triggers the release of interleukin (IL)-1β and IL-18 [35,36]. Additionally, sex hormones, particularly oestrogen, influence mitochondrial RS production [37].

Mitochondria are the principal source of cellular RS, including superoxide and hydrogen peroxide, and play a crucial role in immune responses to infection and tissue damage [38]. Although mitochondrial RS were once considered harmful metabolic byproducts, they are now recognized as essential regulators of immune function [39]. The interplay between mitochondrial RS and immune processes is vital for the activation of immune receptors, such as Toll-like receptors (TLRs), which drive inflammation and immune cell recruitment [40]. However, excessive RS levels can damage cellular components, leading to chronic inflammation, autoimmunity, and impaired immune responses [41]. In particular, neutrophil activity, essential for pathogen detection, is influenced by cellular redox balance [42]. An imbalance in RS can impair phagocytosis, reduce neutrophil extracellular trap (NET) formation, and alter immune defense [43].

### 3.2. Redox Signaling in Tissue Repair and Fibrosis

Redox signaling plays a pivotal role in tissue repair and wound healing, where reactive species are involved in cellular responses to injury. Neutrophils and macrophages are recruited to the wound site and generate RS to eliminate invading pathogens [44]. Meanwhile, fibroblasts, keratinocytes, and endothelial cells regulate lower levels of RS production to support tissue regeneration [45]. Hydrogen peroxide, produced at the wound site, acts as a redox signaling molecule, facilitating tissue repair and preventing infection [46]. The process also triggers a haemostatic response, with platelet activation and the initiation of the coagulation cascade leading to the release of thrombin, which stimulates NOX-dependent RS production in various cell types [47]. Notably, inhibiting NOX activity in vascular smooth muscle cells can block the mitogenic effects of thrombin, underscoring the importance of NOX in cell proliferation and tissue repair [48].

In fibrosis, excessive or prolonged RS production impairs the resolution of inflammation and disrupts normal tissue repair mechanisms, leading to excessive ECM deposition. NOX enzymes, responsible for RS generation, play a crucial role in fibrotic diseases across multiple organs [49,50]. In liver fibrosis, hepatic stellate cells (HSCs) become activated and are the primary producers of collagen and other ECM components. NOX activity is essential for the activation and proliferation of HSCs, and chronic liver injury, such as hepatotoxic or cholestatic conditions, drives fibrosis via RS signaling [51]. Similarly, in renal fibrosis, NOX-derived RS enhance mesangial cell proliferation and the production of TGF-β, a key mediator of fibrosis [52]. In cardiac fibrosis, NOX enzymes contribute to post-myocardial infarction repair but also impair heart function by promoting excessive ECM deposition [53].

### 3.3. Redox Modulation in Fibrosis Development and Progression

Fibrosis is a complex, multi-step process involving the accumulation of ECM components, leading to tissue scarring and organ dysfunction. Cytokines and growth factors create a profibrotic microenvironment, promoting fibroblast activation and ECM production [54]. A regulatory loop involving chemokines and TGF-β has been identified, where ECM components store TGF-β in a latent form [55]. Upon activation, TGF-β drives ECM deposition, inducing collagen, fibronectin, and proteoglycan production [56]. Elevated TGF-β levels are associated with increased fibrosis severity, as seen in conditions such as liver fibrosis, renal fibrosis, and cardiac fibrosis [57]. Interestingly, the role of oxidative and reductive stress in fibrotic progression is interdependent. While RS-driven oxidative stress exacerbates fibrosis by promoting inflammation and tissue damage, reductive stress hampers normal tissue repair, further exacerbating ECM deposition and fibrosis [58,59]. A deeper understanding of how redox pathways modulate these fibrotic processes could open up new therapeutic avenues for preventing and treating fibrotic diseases.

## 4. Biology of Insulin-like Growth Factors (IGFs) and IGF-Binding Proteins (IGFBPs)

### 4.1. Overview of the IGF System

The insulin-like growth factor (IGF) system includes two key polypeptides, IGF-1 and IGF-2, which share structural homology with insulin. These factors interact with cell surface receptors and binding proteins to regulate cellular growth, proliferation, differentiation, survival against apoptosis, and migration. The IGF system plays a critical role in various physiological and pathological conditions, including cancer, diabetes, atherosclerosis, and neurodegeneration [60].

IGF-1 and IGF-2 exert their effects by binding to receptors from the insulin/IGF family, including

- IGF-1 receptor (IGF-1R);
- Insulin receptor isoform A (IR-A) and insulin receptor isoform B (IR-B);
- IGF-2 receptor (IGF-2R).

Since the late 1960s, researchers have recognized proteins capable of binding IGFs with high affinity [61]. By 1991, six IGFBPs (IGFBP-1 to IGFBP-6) had been identified in biological fluids, with their genes cloned and analyzed [62]. IGFBPs are 24–40 kDa proteins that share structural features, including

- N-terminal domain, with high cysteine content, crucial for IGF binding;
- C-terminal domain, with similar structure, also involved in IGF binding;
- Middle linker domain, less conserved, subject to post-translational modifications like glycosylation and phosphorylation [63].

IGFBPs are expressed in several tissues and modulate IGF activity through both endocrine and autocrine/paracrine mechanisms. IGFBPs were initially thought to solely inhibit IGF activity, but recent findings suggest they can also enhance IGF actions in certain circumstances [64].

IGF and insulin genes originated from a common ancestor approximately 500–600 million years ago. The liver is the primary producer of circulating IGFs, though they are also found in other tissues where they act locally. IGF-1 and IGF-2 are single-chain polypeptides consisting of 70 and 67 amino acids, respectively, and share structural similarities with insulin [60].

IGF-1 expression is primarily regulated by growth hormone (GH) and plays a key role in mediating its effects, as described in the somatomedin hypothesis [65]. IGF-2 is important for placental and embryonic development and is most abundant during early life [66].

### 4.2. Biological Functions and Signaling Pathways

IGFs exert diverse cellular effects, including

- Proliferation: IGFs stimulate cell division mainly through the Ras/MAPK pathway.
- Survival: IGFs prevent apoptosis via the PI3K/Akt pathway.
- Differentiation and migration: IGFs enhance differentiation in myoblasts and promote migration in normal and cancer cells.
- Metabolic effects: IGFs exhibit insulin-like effects, such as increasing glucose uptake in adipocytes and muscle cells. IGF therapy for diabetes was largely discontinued due to concerns about worsening diabetic retinopathy [61,64].

There is substantial evidence linking disruptions in the IGF system to cancer. Studies show that elevated IGF-I levels correlate with increased risk for several malignancies, including prostate, colorectal, and breast cancer [67].

IGFs contribute to

- Tumor growth and survival;
- Cell migration and invasion;
- Tumor angiogenesis, particularly under hypoxic conditions, via stimulating vascular endothelial growth factor (VEGF) production.

Animal studies confirm that reduced IGF-1 levels are associated with decreased tumor growth, further supporting the role of IGFs in cancer progression [68].

IGFBPs regulate various cellular functions, including proliferation, survival, differentiation, migration, and invasion (Table 1). More recently, they have also been implicated in key biological processes such as senescence, autophagy, and angiogenesis. Due to these roles, IGFBPs contribute to several physiological and pathological processes, including metabolism, immune function, cancer, and neurological disorders [69].

IGFBP-1 binds IGF-1 and IGF-2 with equal affinity and can either inhibit or enhance their activity. Its effect is dependent on phosphorylation: when phosphorylated on serine residues in the linker domain, IGFBP-1 binds IGFs more tightly, leading to inhibition. In contrast, the non-phosphorylated form exhibits lower affinity and is associated with enhanced IGF activity [70].

IGFBP-2 binds both IGF-1 and IGF-2, with a slight preference for IGF-2. Unlike other IGFBPs, it is neither glycosylated nor phosphorylated. In vitro studies have shown that IGFBP-2 broadly inhibits IGF activity. Similar to IGFBP-1, it contains an Arg-Gly-Asp (RGD) motif in its C-terminal domain, allowing for interaction with α5β1 and αVβ3 integrins. This interaction facilitates cell association and enables IGF-independent functions [71].

IGFBP-3 binds IGF-1 and IGF-2 with equal affinity and primarily inhibits IGF activity in various cell types [72]. However, some studies suggest that IGFBP-3 can also enhance IGF effects, depending on its interaction with cells [73]. IGFBP-3 undergoes N-glycosylation and may also be phosphorylated. While glycosylation reduces its ability to associate with cells, it does not impact its binding to IGFs. Phosphorylation, however, inhibits cell association and reduces IGF-binding affinity [74].

IGFBP-4 also binds IGF-1 and IGF-2 with equal affinity and undergoes N-glycosylation, which does not affect its ability to bind IGFs [75]. In vitro, IGFBP-4 primarily acts as an IGF inhibitor. For instance, studies found that IGFBP-4 secreted by human mesenchymal stem cells suppresses the IGF-induced formation of regulatory T-lymphocytes [76].

Approximately half of circulating IGFBP-5 exists in ternary complexes with IGFs and the acid-labile subunit, while the remainder exists in binary complexes with IGFs or remains unbound. IGFBP-5 has a slight preference for binding IGF-2 over IGF-1 [77]. O-glycosylation and phosphorylation of IGFBP-5 reduce its ability to bind heparin but do not impact its affinity for IGFs or the acid-labile subunit [78].

Unlike other IGFBPs, IGFBP-6 exhibits a ~50-fold higher affinity for IGF-2 than for IGF-1. Consequently, IGFBP-6 primarily inhibits IGF-2’s effects on cell proliferation, differentiation, migration, and survival in vitro [2]. IGFBP-6 is O-glycosylated in its linker domain, reducing its ability to associate with cells and extending its circulating half-life, without affecting its affinity for IGFs [78].

A more recent addition to the IGFBP family, IGFBP-7, is a 30 kDa secreted glycoprotein. Although it shares the ability to bind IGFs, IGFBP-7 has only 20–25% sequence similarity with other IGFBPs and binds IGFswith approximately 100 times lower affinity than other family members. IGFBP-7 is produced in various tissues and cell types, including peripheral nerves, smooth muscle cells, and epithelial cells. However, it is not expressed in lymphocytes, plasma cells, or adipocytes. Additionally, IGFBP-7 expression levels vary within the same cell type, such as kidney epithelial cells, depending on their location within the organ. This suggests that IGFBP-7 may have specialized tissue-specific roles [79].

**Table 1:** Localization and actions of IGFBP isoforms.

Isoform	Expression and Localization	Actions	Refs.
IGFBP-1	Many organs, with high expression in liver and pancreas	- Inhibits or enhances IGFs actions; - Increases cell migration; - Stimulates osteoclast differentiation and bone degradation; - Regulates placental and fetal growth; - Acts on insulin metabolism.	[70]
IGFBP-2	Liver, adipocytes, and the reproductive and central nervous systems	- Preferentially binds IGF-2 and inhibits IGFs actions; - Increases cancer cell proliferation, survival and migration/invasion; - Regulates glucose homeostasis.	[71]
IGFBP-3	Many organs, with high expression in liver and kidney	- Inhibits IGFs actions; - Promotes senescence; - Tumor suppression and pro-tumorigenic actions; - Modulates angiogenesis; - Regulates stem cell biology; - Influences pre- and postnatal growth.	[73,74]
IGFBP-4	Liver, kidney, and uterus	- Inhibits IGF actions; - Regulates bone growth; - Regulates adult skeletal growth; - Induces cardiomyocyte differentiation; - Inhibits angiogenesis.	[75]
IGFBP-5	Bone, lung, kidney, mammary glands, testis, ovary, uterus, and placenta	- Preferentially binds IGF-2; - Increases fibrosis; - Inhibited prenatal growth; - Regulates muscle development; - Decreases female fertility.	[77,78]
IGFBP-6	Many organs, with high expression in prostate, cervix, mammary tissue, and adipose tissue	- Preferentially binds IGF-2; - Inhibits cell proliferation, differentiation, migration and survival; - Inhibits apoptosis; - Inhibits basal and VEGF-induced angiogenesis; - Promotes immune system activation.	[3,80]
IGFBP-7	Many organs, with high expression in brain, liver, pancreas, and skeletal muscle	- Regulates angiogenesis, apoptosis; - Regulates cell growth and progression in cancer.	[79]

Abbreviations: IGFBP, insulin-like growth factor binding protein; IGF, insulin-like growth factor; VEGF, vascular endothelial growth factor.

### 4.3. IGFBPs in Tissue Repair and Regeneration

IGFBPs play a significant role in fibrosis, with IGFBP-7 and TGF-β jointly regulating HSCs to accelerate fibrosis progression.

- Liver fibrosis: In chronic hepatitis C, increased production of IGFBPs has been linked to fibrosis severity [81].
- Primary myelofibrosis (PMF): Elevated IGFBP-2 levels have been observed in PMF patients compared to healthy individuals, suggesting a potential role in disease progression [82].
- IGFBP-6 and microenvironmental alterations: IGFBP-6 regulates fibroblast proliferation and senescence and is involved in the Sonic Hedgehog (SHH)/Toll-like receptor 4 axis in PMF. Studies have shown that IGFBP-6 and SHH activators promote mesenchymal stromal cell differentiation while upregulating cancer-associated fibroblast markers. Inhibition of the SHH pathway reverses these effects, confirming its involvement in TLR4 activation and microenvironmental alterations [83].

IGFBP-6 plays a crucial role in tissue repair and remodeling, fibrosis, and immune regulation. Various studies have highlighted the involvement of IGFBPs in fibrosis progression, suggesting their potential as diagnostic biomarkers or therapeutic targets. IGFBP-6 expression levels vary across different fibrotic conditions, including dermal, renal, hepatic, cardiac fibrosis, and myelofibrosis [8].

## 5. IGFBP-6 and Redox Biology: A Potential Connection

### 5.1. Biology and Functions of IGFBP-6

IGFBP-6 is a secreted protein composed of 216 amino acids, organized into three distinct domains. It is widely expressed in various tissues, including the lungs, liver, gastrointestinal tract, and central nervous system. Its expression is regulated by multiple physiological factors such as cAMP, IGFs, retinoic acid, vitamin D, p53, and glucocorticoids. Additionally, signaling pathways like Wnt and Hedgehog contribute to its regulation [2]. IGFBP-6 has been implicated in human diseases, particularly in cancer biology and fibrosis. There are well-established relationships between immune responses, stromal activity, and fibrosis, which serve as prognostic markers and predictors of response to cancer immunotherapy [8]. In primary myelofibrosis, a significant increase in IGFBP-6 expression levels was observed in patients, whereas those harboring the JAK2V617F mutation exhibited near-normal levels [83].

**Figure 2:**
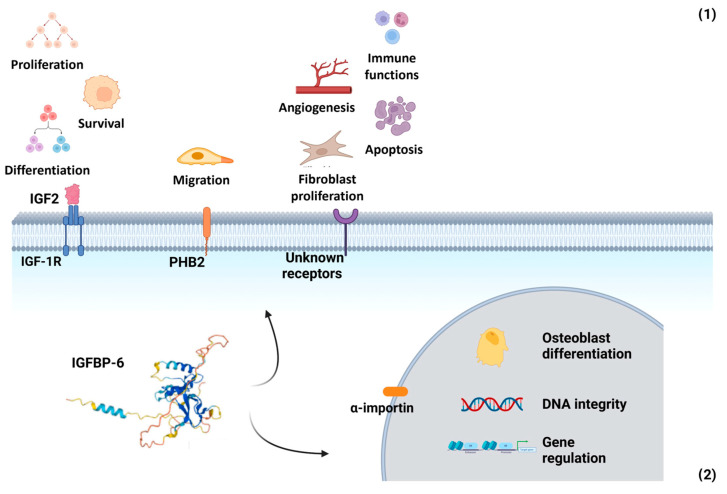
Multifaceted roles of IGFBP-6 in extracellular and nuclear compartments. (**1**) Extracellular functions: IGFBP-6 exerts both IGF-dependent and IGF-independent actions. In its IGF-dependent role, it binds IGF-2 with high affinity, inhibiting IGF-2-mediated cell proliferation, differentiation, migration, and survival. Independently of IGFs, IGFBP-6 modulates cell migration via interaction with prohibitin-2 (PHB2) and may also interact with as-yet-unidentified cell surface receptors. These interactions influence angiogenesis, fibroblast proliferation, apoptosis, and immune function by promoting chemotaxis of T cells, monocytes, and neutrophils, as well as enhancing neutrophil oxidative burst. (**2**) Nuclear functions: IGFBP-6 can translocate to the nucleus via an α-importin-dependent mechanism. Once inside the nucleus, it may regulate gene expression, influence osteoblast differentiation, and participate in the maintenance of DNA integrity. Created with BioRender.com.

IGFBP-6 exerts both IGF-dependent and IGF-independent effects (Figure 2):

- IGF-dependent actions: IGFBP-6 preferentially binds IGF-2, inhibiting its ability to promote cell proliferation, differentiation, migration, and survival. It also facilitates IGF-2 binding to the IGF type 2 receptor (IGF-IIR/CI-MPR), leading to IGF-2 internalization and degradation [63].
- IGF-independent actions: IGFBP-6 modulates cell migration through its interaction with prohibitin-2 (PHB2), independently of IGF-2 binding. Additionally, it influences angiogenesis, fibroblast proliferation, and apoptosis, though the receptors mediating these immune-related effects remain unidentified [84,85].

IGFBP-6 can be transported into the nucleus via an α-importin-dependent mechanism, where it interacts with the vitamin D receptor (VDR). This interaction disrupts the retinoid X receptor (RXR)/VDR transcription complex. Additionally, IGFBP-6 has been shown to associate with nuclear proteins, including thyroid hormone receptor-α1 (TRα1) and histone H2Br. It also binds to the promoter region of the early growth response-1 (EGR-1) gene, suggesting a role in gene regulation. In the cytoplasm, IGFBP-6 regulates the nuclear import of Ku80, a protein involved in non-homologous end joining repair (NHEJR), a key DNA repair pathway [80].

When dendritic cells (DCs) are exposed to 39 °C, they undergo apoptosis and necrosis, leading to an accumulation of IGFBP-6 in the conditioned medium after 48 h. IGFBP-6 plays a functional role in response to hyperthermia, promoting the chemotaxis of monocytes and T lymphocytes, but not B lymphocytes [86].

### 5.2. Redox Homeostasis and IGFBP-6: A Dynamic Interplay

Recent studies have highlighted the potential role of IGFBP-6 in modulating redox biology, particularly in the context of oxidative stress and inflammation. IGFBP-6 has been suggested to interact with redox-sensitive signaling pathways, influencing cellular responses to oxidative stress, immune cell activation, and tissue repair processes. These actions extend beyond its IGF-2-related effects and suggest a broader functional role in redox-regulated biological systems [3].

The interplay between IGFBP-6 and redox homeostasis represents a complex regulatory network, where IGFBP-6 both influences and responds to oxidative stress, shaping cellular functions such as immune response, proliferation, differentiation, and survival. One example of IGFBP-6’s involvement in redox regulation is its response to hyperthermia. When exposed to hyperthermic conditions, IGFBP-6 is rapidly secreted in microvesicles and exosomes, with secretion occurring within three hours. Interestingly, IGFBP-6 has been detected in the conditioned medium of monocyte-derived DCs exposed to both hyperthermia and hydrogen peroxide. This finding suggests that mild oxidative stress may serve as a trigger for immune cell communication through IGFBP-6 and extracellular vesicles (EVs), highlighting its role in immune modulation and redox homeostasis [87].

IGFBP-6 has been implicated in neutrophil activation during inflammation, specifically in oxidative burst, granule release, and chemotaxis. A study demonstrated that when neutrophils were exposed to IGFBP-6 for 50 min, there was a significant increase in ROS production, with peak ROS levels observed at a concentration of 1 μg/mL of IGFBP-6. These effects are indicative of IGFBP-6 functioning as a priming factor that enhances NADPH oxidase activity and promotes redox-dependent neutrophil effector functions. These results indicate that IGFBP-6 acts as an activator of neutrophil responses, potentially enhancing the immune system’s ability to respond to infections and tissue damage through redox signaling by amplifying ROS-mediated antimicrobial activity and innate immune responses [88].

Mechanistically, IGFBP-6 has been shown to influence mitochondrial metabolism in tumor cells. In MDA-231 breast cancer cells, IGFBP-6 modulates lactate signaling through G protein-coupled receptor 81 (GPR81) and upregulates genes related to mitochondrial biogenesis and oxidative phosphorylation [89]. This metabolic reprogramming is accompanied by increased expression of antioxidant genes such as heme oxygenase 1, enhancing cellular resilience to oxidative stress [89]. These effects may support tumor cell survival and adaptation in oxidative microenvironments, potentially promoting tumor progression and chemoresistance.

In the context of fibrosis, there is growing evidence that IGFBP-6 may modulate fibroblast behavior under redox-influenced conditions. In primary myelofibrosis, IGFBP-6 has been implicated in the activation of the Sonic Hedgehog (SHH)/Toll-like receptor 4 (TLR4) axis, promoting stromal reprogramming and fibroblast proliferation [83]. Both SHH and TLR4 signaling are known to be modulated by redox cues, including mitochondrial ROS and NADPH oxidase-derived species. Moreover, IGFBP-6 expression is induced in fibroblasts exposed to oxidative stimuli such as H_2_O_2_, suggesting that its upregulation may be part of a pro-fibrotic, redox-sensitive regulatory circuit. While animal model data are currently lacking, these findings provide cellular-level evidence linking IGFBP-6 to redox-dependent fibrogenesis and warrant further investigation into its mechanistic role in tissue remodeling and fibrosis progression.

IGFBP-6 is also involved in cellular senescence, a process strongly influenced by oxidative stress and inflammation. In fibroblasts undergoing senescence, exposure to hydrogen peroxide or physiological oxygen levels resulted in increased expression of IGFBP-6 [3]. This response is associated with increased senescence markers and may contribute to the senescence-associated secretory phenotype (SASP), thereby sustaining chronic inflammation and fibrotic remodeling. While the specific pathways remain under investigation, this suggests that IGFBP-6 expression may be regulated by ROS-sensitive transcription factors and redox-modulated chromatin remodeling mechanisms.

Neuroprotective properties of IGFBP-6 have also been demonstrated in oxidative stress models. In primary cortical neurons treated with human mesenchymal stem cell-conditioned medium (hMSC-CM) enriched in IGFBP-6, protection against H_2_O_2_-induced damage was observed. This effect was mediated via the activation of the PI3K/Akt and IGF-1R signaling pathways, both of which are known to counteract oxidative stress by promoting cell survival, inhibiting apoptosis, and stimulating antioxidant responses [90]. The engagement of IGF-1R in this context suggests that IGFBP-6 may act not only independently but also synergistically with IGF signaling under oxidative stress.

**Figure 3:**
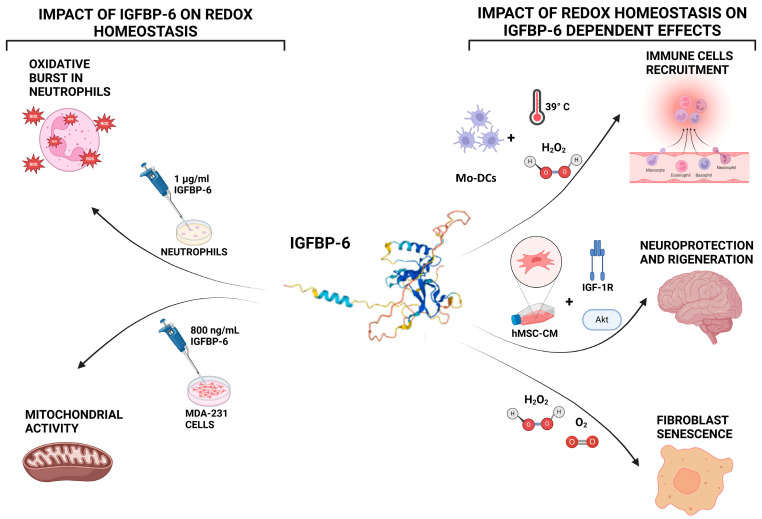
Bidirectional interactions between IGFBP-6 and redox homeostasis. Left panel: IGFBP-6 influences redox biology by stimulating the production of reactive oxygen species (ROS) in neutrophils, with maximal ROS generation observed at 1 μg/mL after 50 min of exposure. In MDA-MB-231 breast cancer cells, IGFBP-6 treatment enhances mitochondrial metabolism and upregulates genes related to oxidative phosphorylation and antioxidant defenses. Right panel: Redox conditions also regulate IGFBP-6 activity. Hyperthermia or hydrogen peroxide (H_2_O_2_) exposure induces IGFBP-6 expression in monocyte-derived dendritic cells (DCs). In fibroblasts, oxidative stress or physiological oxygen tension elevates IGFBP-6 levels during cellular senescence. In neurons, IGFBP-6 contributes to cell survival under oxidative conditions through activation of the PI3K/Akt and IGF-1R signaling pathways. Created with BioRender.com.

These findings further emphasize the importance of IGFBP-6 in maintaining redox homeostasis, particularly in protecting cells from oxidative damage and supporting tissue repair in the nervous system [90] (Figure 3).

These observations position IGFBP-6 at the intersection of several redox-sensitive biological processes described earlier in this review. As detailed in Section 3, redox signaling regulates inflammation, immune defense, tissue remodeling, and tumor progression. IGFBP-6 contributes to these same processes through multiple pathways: it amplifies neutrophil oxidative burst during inflammation; promotes fibroblast senescence and proliferation under oxidative stress, contributing to fibrotic microenvironments; and enhances mitochondrial metabolism and antioxidant capacity in cancer cells, potentially supporting tumor survival and progression. By integrating redox regulation with immune modulation, fibrogenesis, and oncogenesis, IGFBP-6 emerges as a functionally versatile mediator whose biological effects mirror and potentially magnify broader redox-driven pathophysiological mechanisms. This convergence underlines the need to further investigate IGFBP-6 as a nodal point within redox signaling networks in disease. Collectively, IGFBP-6 emerges as a redox-responsive modulator involved in key processes such as neutrophil activation, mitochondrial reprogramming, fibroblast senescence, and neuronal survival. Its ability to engage pathways like PI3K/Akt, IGF-1R, and mitochondrial biogenesis links IGFBP-6 to critical outcomes in immune regulation, cancer progression, and fibrotic tissue remodeling. Despite these insights, the precise molecular mechanisms and receptors involved remain incompletely defined, highlighting an important direction for future investigation.

## 6. Conclusions

This review highlights emerging evidence that IGFBP-6 functions as a redox-sensitive modulator of immune activation, fibrosis, and tumor biology, acting through both IGF-dependent and IGF-independent mechanisms. IGFBP-6 enhances oxidative burst in neutrophils, contributes to fibroblast senescence and activation under oxidative stress, and reprograms mitochondrial metabolism and antioxidant responses in tumor cells. These actions align closely with known redox-regulated pathways discussed earlier in the review, underscoring the protein’s relevance in oxidative stress-related pathologies.

Despite these advances, several limitations remain. Most studies to date have been conducted in vitro, and direct evidence from animal models linking IGFBP-6 to redox-modulated fibrotic or oncogenic phenotypes is limited. Furthermore, the specific receptors and signaling intermediates mediating IGFBP-6’s redox-related effects remain largely unidentified.

Looking ahead, future research should prioritize mechanistic studies to identify the receptors and signaling pathways involved in IGFBP-6-mediated redox responses. Additionally, in vivo models of inflammation, fibrosis, and cancer are needed to validate the relevance of IGFBP-6 as a therapeutic target. Given its multifaceted roles at the intersection of redox biology, immune regulation, and tissue remodeling, IGFBP-6 holds significant potential for advancing our understanding of redox-dependent disease processes and informing new therapeutic strategies.

## Data Availability

Not applicable.

## Abbreviations

The following abbreviations are used in this manuscript:

IGFBP-6	Insulin-like growth factor-binding protein 6
IGF-2	Insulin-like growth factor 2
RS	Reactive species
IGFs	Insulin-like growth factors
IGFBPs	Growth factor-binding proteins
ROS	Reactive oxygen species
RNS	Reactive nitrogen species
O_2_^−^	Superoxide anion
NOXs	NADPH oxidases
UV	Ultraviolet
SOD	Superoxide dismutase
H_2_O_2_	Hydrogen peroxide
NF-κB	Nuclear factor-kappa B
AP-1	Activator protein-1
MAPK	Mitogen-activated protein kinase
TGF-β	Tumor growth factor-β
ECM	Extracellular matrix
TNF	Tumor necrosis factor
NLRP3	NLR pyrin domain-containing 3
IL	Interleukin
TLRs	Toll-like receptors
NET	Neutrophil extracellular trap
HSCs	Hepatic stellate cells
IGF-1R	Insulin-like growth factor receptor 1
IGF-2R	Insulin-like growth factor receptor 2
IR-A	Insulin receptor isoform A
IR-B	Insulin receptor isoform B
GH	Growth hormone
VEGF	Vascular endothelial growth factor
SHH	Sonic hedgehog
PHB2	Prohibitin-2
VDR	Vitamin D receptor
RXR	Retinoid X receptor
TRα1	Thyroid hormone receptor-α1
EGR-1	Early growth response-1
NHEJR	Non-homologous end joining repair
DCs	Dendritic cells
GPR81	G protein-coupled receptor 81
hMSC-CM	Human mesenchymal stem cell-conditioned medium

## References

[1] LeRoith D., Holly J.M.P., Forbes B.E. (2021). Insulin-like Growth Factors: Ligands, Binding Proteins, and Receptors. Mol. Metab..

[2] Bach L.A. (2016). Current Ideas on the Biology of IGFBP-6: More than an IGF-II Inhibitor?. Growth Horm IGF Res..

[3] Bach L.A. (2015). Recent Insights into the Actions of IGFBP-6. J. Cell Commun. Signal.

[4] Foyer C.H., Noctor G. (2005). Redox Homeostasis and Antioxidant Signaling: A Metabolic Interface between Stress Perception and Physiological Responses. Plant Cell.

[5] Aranda-Rivera A.K., Cruz-Gregorio A., Arancibia-Hernández Y.L., Hernández-Cruz E.Y., Pedraza-Chaverri J. (2022). RONS and Oxidative Stress: An Overview of Basic Concepts. Oxygen.

[6] Chandimali N., Bak S.G., Park E.H., Lim H.-J., Won Y.-S., Kim E.-K., Park S.-I., Lee S.J. (2025). Free Radicals and Their Impact on Health and Antioxidant Defenses: A Review. Cell Death Discov..

[7] Li B., Ming H., Qin S., Nice E.C., Dong J., Du Z., Huang C. (2025). Redox Regulation: Mechanisms, Biology and Therapeutic Targets in Diseases. Signal Transduct. Target. Ther..

[8] Liso A., Venuto S., Coda A.R.D., Giallongo C., Alberto Palumbo G., Tibullo D. (2022). IGFBP-6: At the Crossroads of Immunity, Tissue Repair and Fibrosis. Int. J. Mol. Sci..

[9] Remigante A., Cordaro M., Morabito R. (2024). Redox Homeostasis and Antioxidant Strategies in the Pathophysiology. Antioxidants.

[10] Hong Y., Boiti A., Vallone D., Foulkes N.S. (2024). Reactive Oxygen Species Signaling and Oxidative Stress: Transcriptional Regulation and Evolution. Antioxidants.

[11] Zorov D.B., Juhaszova M., Sollott S.J. (2014). Mitochondrial Reactive Oxygen Species (ROS) and ROS-Induced ROS Release. Physiol. Rev..

[12] Cho K.J., Seo J.M., Kim J.H. (2011). Bioactive Lipoxygenase Metabolites Stimulation of NADPH Oxidases and Reactive Oxygen Species. Mol. Cells.

[13] Cipriano A., Viviano M., Feoli A., Milite C., Sarno G., Castellano S., Sbardella G. (2023). NADPH Oxidases: From Molecular Mechanisms to Current Inhibitors. J. Med. Chem..

[14] Jomova K., Raptova R., Alomar S.Y., Alwasel S.H., Nepovimova E., Kuca K., Valko M. (2023). Reactive Oxygen Species, Toxicity, Oxidative Stress, and Antioxidants: Chronic Diseases and Aging. Arch. Toxicol..

[15] Zhao Y., Ye X., Xiong Z., Ihsan A., Ares I., Martínez M., Lopez-Torres B., Martínez-Larrañaga M.R., Anadón A., Wang X. (2023). Cancer Metabolism: The Role of ROS in DNA Damage and Induction of Apoptosis in Cancer Cells. Metabolites.

[16] Iqbal M.J., Kabeer A., Abbas Z., Siddiqui H.A., Calina D., Sharifi-Rad J., Cho W.C. (2024). Interplay of Oxidative Stress, Cellular Communication and Signaling Pathways in Cancer. Cell Commun. Signal.

[17] Jomova K., Alomar S.Y., Alwasel S.H., Nepovimova E., Kuca K., Valko M. (2024). Several Lines of Antioxidant Defense against Oxidative Stress: Antioxidant Enzymes, Nanomaterials with Multiple Enzyme-Mimicking Activities, and Low-Molecular-Weight Antioxidants. Arch. Toxicol..

[18] Wang Y., Branicky R., Noë A., Hekimi S. (2018). Superoxide Dismutases: Dual Roles in Controlling ROS Damage and Regulating ROS Signaling. J. Cell Biol..

[19] Kurutas E.B. (2016). The Importance of Antioxidants Which Play the Role in Cellular Response against Oxidative/Nitrosative Stress: Current State. Nutr. J..

[20] López-Grueso M.J., González-Ojeda R., Requejo-Aguilar R., McDonagh B., Fuentes-Almagro C.A., Muntané J., Bárcena J.A., Padilla C.A. (2019). Thioredoxin and Glutaredoxin Regulate Metabolism through Different Multiplex Thiol Switches. Redox Biol..

[21] Berndt C., Lillig C.H., Holmgren A. (2007). Thiol-Based Mechanisms of the Thioredoxin and Glutaredoxin Systems: Implications for Diseases in the Cardiovascular System. Am. J. Physiol. Heart Circ. Physiol..

[22] Seitz R., Tümen D., Kunst C., Heumann P., Schmid S., Kandulski A., Müller M., Gülow K. (2024). Exploring the Thioredoxin System as a Therapeutic Target in Cancer: Mechanisms and Implications. Antioxidants.

[23] Priya Dharshini L.C., Vishnupriya S., Sakthivel K.M., Rasmi R.R. (2020). Oxidative Stress Responsive Transcription Factors in Cellular Signalling Transduction Mechanisms. Cell Signal.

[24] Andrés C.M.C., Pérez de la Lastra J.M., Juan C.A., Plou F.J., Pérez-Lebeña E. (2022). The Role of Reactive Species on Innate Immunity. Vaccines.

[25] Santarpia L., Lippman S.M., El-Naggar A.K. (2012). Targeting the MAPK-RAS-RAF Signaling Pathway in Cancer Therapy. Expert. Opin. Ther. Targets.

[26] Li K., Deng Z., Lei C., Ding X., Li J., Wang C. (2024). The Role of Oxidative Stress in Tumorigenesis and Progression. Cells.

[27] Deng Z., Fan T., Xiao C., Tian H., Zheng Y., Li C., He J. (2024). TGF-β Signaling in Health, Disease, and Therapeutics. Signal Transduct. Target. Ther..

[28] Xiao W., Loscalzo J. (2020). Metabolic Responses to Reductive Stress. Antioxid. Redox Signal..

[29] Zhang S., Wang N., Gao Z., Gao J., Wang X., Xie H., Wang C.-Y., Zhang S. (2025). Reductive Stress: The Key Pathway in Metabolic Disorders Induced by Overnutrition. J. Adv. Res..

[30] Ge M., Papagiannakopoulos T., Bar-Peled L. (2024). Reductive Stress in Cancer: Coming out of the Shadows. Trends Cancer.

[31] Yan L.J. (2014). Pathogenesis of Chronic Hyperglycemia: From Reductive Stress to Oxidative Stress. J. Diabetes Res..

[32] Antar S.A., Ashour N.A., Marawan M.E., Al-Karmalawy A.A. (2023). Fibrosis: Types, Effects, Markers, Mechanisms for Disease Progression, and Its Relation with Oxidative Stress, Immunity, and Inflammation. Int. J. Mol. Sci..

[33] Bhol N.K., Bhanjadeo M.M., Singh A.K., Dash U.C., Ojha R.R., Majhi S., Duttaroy A.K., Jena A.B. (2024). The Interplay between Cytokines, Inflammation, and Antioxidants: Mechanistic Insights and Therapeutic Potentials of Various Antioxidants and Anti-Cytokine Compounds. Biomed. Pharmacother..

[34] Akkız H., Gieseler R.K., Canbay A. (2024). Liver Fibrosis: From Basic Science towards Clinical Progress, Focusing on the Central Role of Hepatic Stellate Cells. Int. J. Mol. Sci..

[35] Morgan M.J., Liu Z.G. (2010). Reactive Oxygen Species in TNFα-Induced Signaling and Cell Death. Mol. Cells.

[36] Kelley N., Jeltema D., Duan Y., He Y. (2019). The NLRP3 Inflammasome: An Overview of Mechanisms of Activation and Regulation. Int. J. Mol. Sci..

[37] Klinge C.M. (2020). Estrogenic Control of Mitochondrial Function. Redox Biol..

[38] Giorgi C., Marchi S., Simoes I.C.M., Ren Z., Morciano G., Perrone M., Patalas-Krawczyk P., Borchard S., Jędrak P., Pierzynowska K. (2018). Mitochondria and Reactive Oxygen Species in Aging and Age-Related Diseases. Int. Rev. Cell Mol. Biol..

[39] Finkel T. (2012). Signal Transduction by Mitochondrial Oxidants. J. Biol. Chem..

[40] Pinegin B., Vorobjeva N., Pashenkov M., Chernyak B. (2018). The Role of Mitochondrial ROS in Antibacterial Immunity. J. Cell Physiol..

[41] Checa J., Aran J.M. (2020). Reactive Oxygen Species: Drivers of Physiological and Pathological Processes. J. Inflamm. Res..

[42] Xie K., Varatnitskaya M., Maghnouj A., Bader V., Winklhofer K.F., Hahn S., Leichert L.I. (2020). Activation Leads to a Significant Shift in the Intracellular Redox Homeostasis of Neutrophil-like Cells. Redox Biol..

[43] Manoj H., Gomes S.M., Thimmappa P.Y., Nagareddy P.R., Jamora C., Joshi M.B. (2025). Cytokine Signalling in Formation of Neutrophil Extracellular Traps: Implications for Health and Diseases. Cytokine Growth Factor. Rev..

[44] De Oliveira S., Rosowski E.E., Huttenlocher A. (2016). Neutrophil Migration in Infection and Wound Repair: Going Forward in Reverse. Nat. Rev. Immunol..

[45] Hunt M., Torres M., Bachar-Wikstrom E., Wikstrom J.D. (2024). Cellular and Molecular Roles of Reactive Oxygen Species in Wound Healing. Commun. Biol..

[46] Zhu G., Wang Q., Lu S., Niu Y. (2017). Hydrogen Peroxide: A Potential Wound Therapeutic Target?. Med. Princ. Pract..

[47] Masselli E., Pozzi G., Vaccarezza M., Mirandola P., Galli D., Vitale M., Carubbi C., Gobbi G. (2020). ROS in Platelet Biology: Functional Aspects and Methodological Insights. Int. J. Mol. Sci..

[48] Burtenshaw D., Hakimjavadi R., Redmond E.M., Cahill P.A. (2017). Nox, Reactive Oxygen Species and Regulation of Vascular Cell Fate. Antioxidants.

[49] Lambeth J.D. (2007). Nox Enzymes, ROS, and Chronic Disease: An Example of Antagonistic Pleiotropy. Free Radic. Biol. Med..

[50] Kato K., Hecker L. (2020). NADPH Oxidases: Pathophysiology and Therapeutic Potential in Age-Associated Pulmonary Fibrosis. Redox Biol..

[51] Liang S., Kisseleva T., Brenner D.A. (2016). The Role of NADPH Oxidases (NOXs) in Liver Fibrosis and the Activation of Myofibroblasts. Front. Physiol..

[52] Sureshbabu A., Muhsin S.A., Choi M.E. (2016). TGF-β Signaling in the Kidney: Profibrotic and Protective Effects. Am. J. Physiol. Renal Physiol..

[53] Liu Z.Y., Liu Z.Y., Lin L.C., Song K., Tu B., Zhang Y., Yang J.J., Zhao J.Y., Tao H. (2024). Redox Homeostasis in Cardiac Fibrosis: Focus on Metal Ion Metabolism. Redox Biol..

[54] Moretti L., Stalfort J., Barker T.H., Abebayehu D. (2022). The Interplay of Fibroblasts, the Extracellular Matrix, and Inflammation in Scar Formation. J. Biol. Chem..

[55] Hyytiäinen M., Penttinen C., Keski-Oja J. (2004). Latent TGF-β Binding Proteins: Extracellular Matrix Association and Roles in TGF-β Activation. Crit. Rev. Clin. Lab. Sci..

[56] Frangogiannis N.G. (2020). Transforming Growth Factor–ß in Tissue Fibrosis. J. Exp. Med..

[57] Pohlers D., Brenmoehl J., Löffler I., Müller C.K., Leipner C., Schultze-Mosgau S., Stallmach A., Kinne R.W., Wolf G. (2009). TGF-β and Fibrosis in Different Organs—Molecular Pathway Imprints. Biochim. Biophys. Acta Mol. Basis Dis..

[58] Richter K., Kietzmann T. (2016). Reactive Oxygen Species and Fibrosis: Further Evidence of a Significant Liaison. Cell Tissue Res..

[59] Manford A.G., Rodríguez-Pérez F., Shih K.Y., Shi Z., Berdan C.A., Choe M., Titov D.V., Nomura D.K., Rape M. (2020). A Cellular Mechanism to Detect and Alleviate Reductive Stress. Cell.

[60] Denley A., Cosgrove L.J., Booker G.W., Wallace J.C., Forbes B.E. (2005). Molecular Interactions of the IGF System. Cytokine Growth Factor Rev..

[61] Bach L.A., Rechler M.M. (1995). Insulin-like Growth Factor Binding Proteins. Diabetes Rev..

[62] Hwa V., Oh Y., Rosenfeld R.G. (1999). The Insulin-like Growth Factor-Binding Protein (IGFBP) Superfamily. Endocr. Rev..

[63] Bach L.A., Fu P., Yang Z. (2013). Insulin-like Growth Factor-Binding Protein-6 and Cancer. Clin. Sci..

[64] Bach L.A. (2018). IGF-Binding Proteins. J. Mol. Endocrinol..

[65] Annunziata M., Granata R., Ghigo E. (2011). The IGF System. Acta Diabetol..

[66] Blyth A.J., Kirk N.S., Forbes B.E. (2020). Understanding IGF-II Action through Insights into Receptor Binding and Activation. Cells.

[67] Pollak M. (2008). Insulin and Insulin-like Growth Factor Signalling in Neoplasia. Nat. Rev. Cancer.

[68] Wu Y., Yakar S., Zhao L., Hennighausen L., LeRoith D. (2002). Circulating Insulin-like Growth Factor-I Levels Regulate Colon Cancer Growth and Metastasis. Cancer Res.

[69] Monzavi R., Cohen P. (2002). IGFs and IGFBPs: Role in Health and Disease. Best. Pract. Res. Clin. Endocrinol. Metab..

[70] Shehab M.A., Iosef C., Wildgruber R., Sardana G., Gupta M.B. (2013). Phosphorylation of IGFBP-1 at Discrete Sites Elicits Variable Effects on IGF-I Receptor Autophosphorylation. Endocrinology.

[71] Qiu Q., Yan X., Bell M., Di J., Tsang B.K., Gruslin A. (2010). Mature IGF-II Prevents the Formation of “Big” IGF-II/IGFBP-2 Complex in the Human Circulation. Growth Horm. IGF Res..

[72] Ricort J.M., Binoux M. (2001). Insulin-like Growth Factor (IGF) Binding Protein-3 Inhibits Type 1 IGF Receptor Activation Independently of Its IGF Binding Affinity. Endocrinology.

[73] Cai Q., Dozmorov M., Oh Y. (2020). Igfbp-3/Igfbp-3 Receptor System as an Anti-Tumor and Anti-Metastatic Signaling in Cancer. Cells.

[74] Conover C.A. (1991). Glycosylation of Insulin-like Growth Factor Binding Protein-3 (IGFBP-3) Is Not Required for Potentiation of IGF-I Action: Evidence for Processing of Cell-Bound IGFBP-3. Endocrinology.

[75] Ning Y., Schuller A.G.P., Conover C.A., Pintar J.E. (2008). Insulin-like Growth Factor (IGF) Binding Protein-4 Is Both a Positive and Negative Regulator of IGF Activity in Vivo. Mol. Endocrinol..

[76] Miyagawa I., Nakayamada S., Nakano K., Yamagata K., Sakata K., Yamaoka K., Tanaka Y. (2017). Induction of Regulatory T Cells and Its Regulation with Insulin-like Growth Factor/Insulin-like Growth Factor Binding Protein-4 by Human Mesenchymal Stem Cells. J. Immunol..

[77] Twigg S.M., Baxter R.C. (1998). Insulin-like Growth Factor (IGF)-Binding Protein 5 Forms an Alternative Ternary Complex with IGFs and the Acid-Labile Subunit. J. Biol. Chem..

[78] Graham M.E., Kilby D.M., Firth S.M., Robinson P.J., Baxter R.C. (2007). The in Vivo Phosphorylation and Glycosylation of Human Insulin-like Growth Factor-Binding Protein-5. Mol. Cell Proteom..

[79] Marinaro J.A., Neumann G.M., Russo V.C., Leeding K.S., Bach L.A. (2000). O-Glycosylation of Insulin-like Growth Factor (IGF) Binding Protein-6 Maintains High IGF-II Binding Affinity by Decreasing Binding to Glycosaminoglycans, and Susceptibility to Proteolysis. Eur. J. Biochem..

[80] Liso A., Capitanio N., Gerli R., Conese M. (2018). From Fever to Immunity: A New Role for IGFBP-6?. J. Cell Mol. Med..

[81] Rosique-Oramas D., Martínez-Castillo M., Raya A., Medina-Ávila Z., Aragón F., Limón-Castillo J., Hernández-Barragán A., Santoyo A., Montalvo-Javé E., Pérez-Hernández J.L. (2020). Production of Insulin-like Growth Factor-Binding Proteins during the Development of Hepatic Fibrosis Due to Chronic Hepatitis C. Rev. Gastroenterol. México.

[82] Ho C.L., Lasho T.L., Butterfield J.H., Tefferi A. (2007). Global Cytokine Analysis in Myeloproliferative Disorders. Leuk. Res..

[83] Longhitano L., Tibullo D., Vicario N., Giallongo C., Spina E.L., Romano A., Lombardo S., Moretti M., Masia F., Coda A.R.D. (2021). IGFBP-6/Sonic Hedgehog/TLR4 Signalling Axis Drives Bone Marrow Fibrotic Transformation in Primary Myelofibrosis. Aging.

[84] Wei Y., Chiang W.C., Sumpter R., Mishra P., Levine B. (2017). Prohibitin 2 Is an Inner Mitochondrial Membrane Mitophagy Receptor. Cell.

[85] Fu P., Yang Z., Bach L.A. (2013). Prohibitin-2 Binding Modulates Insulin-like Growth Factor-Binding Protein-6 (IGFBP-6)-Induced Rhabdomyosarcoma Cell Migration. J. Biol. Chem..

[86] Liso A., Castellani S., Massenzio F., Trotta R., Pucciarini A., Bigerna B., De Luca P., Zoppoli P., Castiglione F., Palumbo M.C. (2017). Human Monocyte-Derived Dendritic Cells Exposed to Hyperthermia Show a Distinct Gene Expression Profile and Selective Upregulation of IGFBP6. Oncotarget.

[87] Conese M., Pace L., Pignataro N., Catucci L., Ambrosi A., Di Gioia S., Tartaglia N., Liso A. (2020). Insulin-like Growth Factor Binding Protein 6 Is Secreted in Extracellular Vesicles upon Hyperthermia and Oxidative Stress in Dendritic Cells but Not in Monocytes. Int. J. Mol. Sci..

[88] Conese M., D’Oria S., Castellani S., Trotta R., Montemurro P., Liso A. (2018). Insulin-like Growth Factor-6 (IGFBP-6) Stimulates Neutrophil Oxidative Burst, Degranulation and Chemotaxis. Inflamm. Res..

[89] Longhitano L., Forte S., Orlando L., Grasso S., Barbato A., Vicario N., Parenti R., Fontana P., Amorini A.M., Lazzarino G. (2022). The Crosstalk between GPR81/IGFBP6 Promotes Breast Cancer Progression by Modulating Lactate Metabolism and Oxidative Stress. Antioxidants.

[90] Jeon H.J., Park J., Shin J.H., Chang M.S. (2017). Insulin-like Growth Factor Binding Protein-6 Released from Human Mesenchymal Stem Cells Confers Neuronal Protection through IGF-1R-Mediated Signaling. Int. J. Mol. Med..

